# Structure of l-arginine and detection of trace dl-arginine by 3D ED

**DOI:** 10.1107/S2053229625005091

**Published:** 2025-06-20

**Authors:** Krishna P. Khakurel, Sohail Mahmoudi

**Affiliations:** a ELI ERIC, ELI Beamlines Facility, Dolni Brezany, Czech Republic; b Vienna Doctoral School in Chemistry (DoSChem), University of Vienna, Währinger Strasse 42, Vienna, 1090, Austria; c Department of Inorganic Chemistry, University of Vienna, Währinger Strasse 42, Vienna, 1090, Austria; University of Delaware, USA

**Keywords:** crystal structure, l-arginine, 3D electron crystallography, 3D ED, trace impurity

## Abstract

3D electron crystallography has emerged as a method with great potential for the structure determination of small mol­ecules and macromolecules com­plementing traditional single-crystal X-ray crystallography and powder X-ray diffraction (PXRD). It offers the unique capability of determining the structures of small mol­ecules and macromolecules from micro- and nanocrystals. In this study, using 3D electron diffraction (3D ED), we determined the single-crystal structure of commercially sourced arginine directly from its bottle. The 3D ED analysis of micro-sized single crystals identified two distinct forms: the l-arginine enanti­omer and the racemic mixture dl-arginine. At the time of writing, neither the Cambridge Structural Database nor the Crystallographic Open Database contain a single-crystal structure of isolated l-arginine (sum formula C_6_H_14_N_4_O_2_), which has been solved in this work by 3D ED. We also present a com­parison of the structures of these mol­ecules solved by 3D ED and PXRD.

## Abstract 2

The single-crystal structures of l-arginine and a trace impurity of racemic dl-arginine present in commercially sourced arginine have been determined using 3D ED. The single-crystal structure for l-arginine was previously unavailable.

## Introduction

X-ray crystallography has made significant contributions to structural chemistry and biochemistry (Deschamps, 2010 ▸). Until recently, a large proportion of deposited structures were predominantly solved using single-crystal X-ray diffraction (SCXRD), which is limited by the size of the crystals required for structure determination (Khakurel *et al.*, 2019 ▸).

Often, for many mol­ecules, growing large crystals of the quality necessary for single-crystal X-ray crystallography is a cumbersome process. When solving single-crystal structures using SCXRD has been challenging, powder X-ray diffraction (PXRD) has served as an alternative method for determining the crystal structure (Harris *et al.*, 2001 ▸; Harris, 2012 ▸; Courvoisier *et al.*, 2012 ▸; Williams *et al.*, 2015 ▸). PXRD has been widely used in the screening of polymorphic forms in chemistry and pharmaceutical research (Spiliopoulou *et al.*, 2020 ▸). However, PXRD often presents several challenges in solving structures. Proper indexing of the powder diffraction data not only demands a high degree of crystallinity, but also needs purity of the sample ensuring that no mixture of other phases is present (Habermehl *et al.*, 2014 ▸). Moreover, the structures solved by PXRD are likely to be less accurate than those solved by single-crystal methods (Pan *et al.*, 2012 ▸). Advances in combining PXRD with machine-learning tools to simplify the refinement problem and solve structures from low-quality data is underway (Niitsu *et al.*, 2024 ▸). However, this does not solve the inherent problems associated with PXRD. Furthermore, the absence of a standardized process for evaluating and validating structural models makes PXRD less attractive for cases where single-crystal structures can be determined using methods such as SCXRD and 3D ED.

PXRD is a bulk measurement technique and can differentiate the forms of the analyte. However, determination of the structures of multiple forms of organic com­pounds in the same experiment is often challenging. Furthermore, with X-ray-based techniques, accurate determination of the precise positions of the H atoms in organic com­pounds has been a significant challenge and often needs additional processing of the data. This information is provided by 3D ED with standard data processing.

In the past decade, 3D ED has em­erged as a method with huge potential for solving the structures of mol­ecules from submicro/nanocrystals (Gemmi *et al.*, 2019 ▸; Gruene *et al.*, 2018 ▸). The method has evolved over the years and is now routinely used in solving the structures of mol­ecules from micro/nanocrystals which otherwise would not be possible with SCXRD. Apart from the possibility of solving structures from tiny nano/micro-sized crystals, 3D ED also offers additional advantages over X-ray crystallography, such as in locating the precise position of H atoms (Palatinus *et al.*, 2017 ▸) and the possibility to model atomic partial charges (Yonekura *et al.*, 2015 ▸). Among many other developments, the potential of 3D ED to unravel the chirality of a mol­ecule has also been of recent inter­est to the community (Klar *et al.*, 2023 ▸).

One of the striking features of 3D ED, which sets it apart from SCXRD and PXRD, is its sensitivity in determining the structures of the forms and the constituent chemicals in heterogeneous mixtures. The determination of the structure of a chemical com­pound from a heterogeneous mixture has been demonstrated with electron diffraction on a few occasions (Jones *et al.*, 2018 ▸; Gruene *et al.*, 2018 ▸; Unge *et al.*, 2024 ▸). Different conformations of macrocyclic drug com­pounds have been determined previously from the same experiment (Danelius *et al.*, 2023 ▸). In an attempt to solve the single-crystal structure of l-arginine from the commercially purchased 99% pure powder formulation, we determine the single-crystal structure of l-arginine and of the racemate. The single-crystal structure for one of the forms (l-arginine), to the best of our knowledge, has not been reported previously. The work presented here showcases the twofold strength of the 3D ED technique: (i) solving a previously undetermined single-crystal structure and (ii) determining the structure of a trace racemic mixture (<1%) present in a powder.

While the first crystal structure of an amino acid was determined back in 1939, there are several amino acids whose racemic crystal structures are yet to be determined. Among them are asparagine, phenyl­alanine, threonine and lysine (Hughes *et al.*, 2018 ▸). The crystal structure of one of the naturally occurring amino acids, l-arginine, was determined only in the last decade by PXRD (CCDC ID 855058) (Courvoisier *et al.*, 2012 ▸). The long-standing challenge in solving the structure by SCXRD was due to the difficulty in obtaining the size of crystal necessary to collect data *via* SCXRD. We present here the single-crystal structure of l-arginine determined using 3D ED. We com­pare the results with those obtained from PXRD. In the same experiment, we determined the crystal structure of dl-arginine present in a trace amount. The crystal structure of dl-arginine was previously solved using SCXRD (CCDC ID: 152635) (Kingsford-Adaboh *et al.*, 2000 ▸).

## Sample preparation

In order to determine the structure, we used the crystalline powder of l-arginine (99% pure) purchased from Merck (product ID: W381920). The product specification provided by the supplier confirmed the structure using IR spectroscopy. Furthermore, the specification confirms that the foreign insoluble matter present in the sample is <0.005%. The samples were used without any further processing. A suspension was created with hexane to homogeneously spread the crystals over the grid.

## Data collection

**Figure 1:**
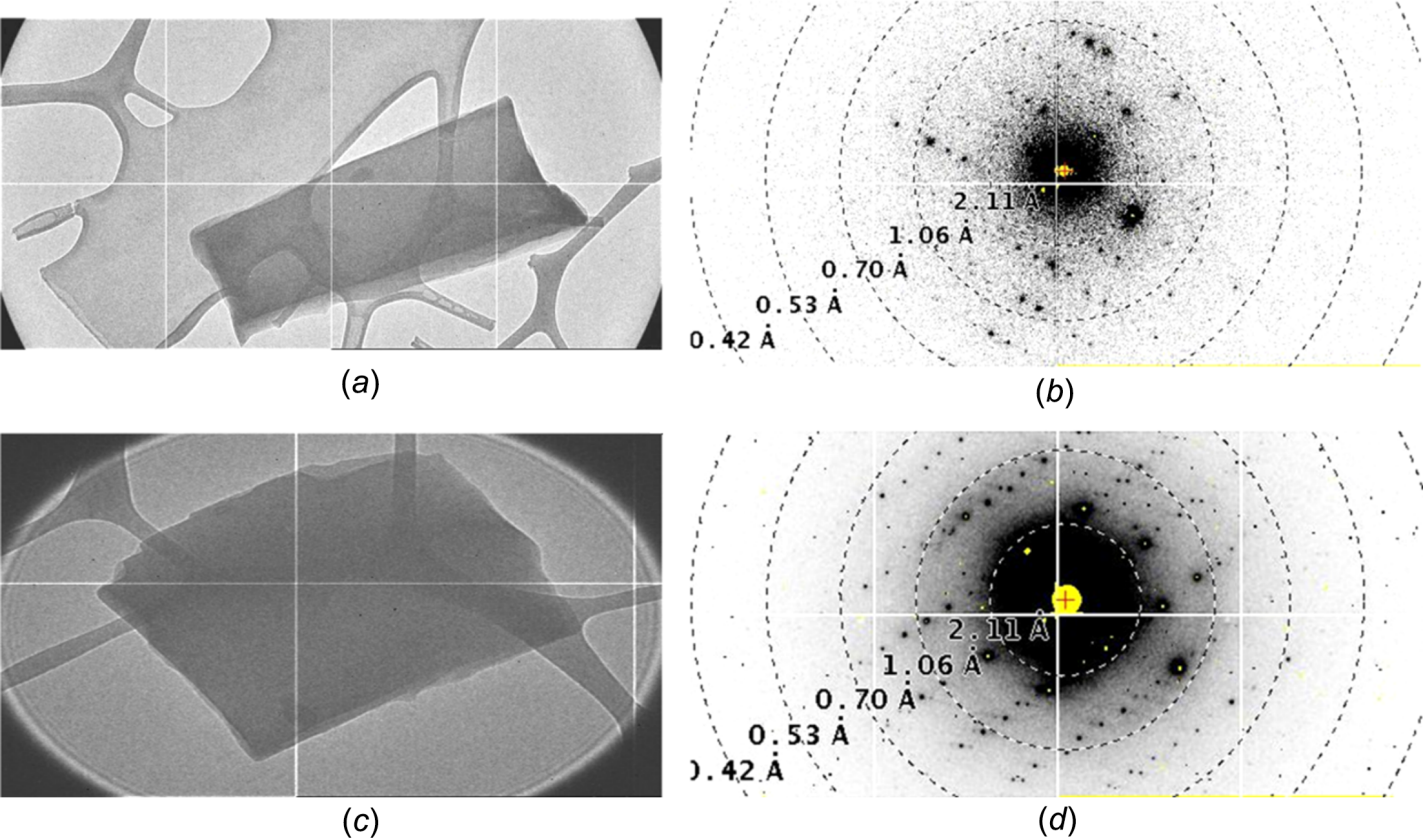
(*a*) An electron micrograph of the dl-arginine crystal. (*b*) One of the diffraction patterns from the dl-arginine crystal. (*c*) Electron micrograph of one of the l-arginine crystals. (*d*) The diffraction pattern from part (*c*).

Data were collected with a JEOL JEM2100Plus, which was equipped with a 512 × 1024 pixel JUNGFRAU detector with a 320 µm thick silicon sensor and a pixel size of 75 µm (Fröjdh *et al.*, 2020 ▸). The Gatan holder ELSA 698 was cooled to −110 °C to reduce radiation damage on the crystal and to prevent the formation of crystal ice at lower tem­per­a­ture. The beam current was confined with a 50 µm condenser lens aperture and spot size 4. This corresponds to a current of about 20 pA. The sample was illuminated with a beam diameter of about 2.2 µm. Data were collected at 1.0°/s and sampled at 10 Hz, *i.e.* 0.1°/frame. Continuous rotation diffraction series were collected from −50 to +70° and from −60 to +70° for the dl-arginine and l-arginine crystals, respectively. The effective detector distance was ∼665 mm which gives a reciprocal pixel size of ∼0.009 Å^−1^. The calibration of the detector distance, the rotation axis and the Jungfrau detector for the 3D ED experiment has been described previously (Fröjdh *et al.*, 2020 ▸). The data collection was carried out using in-house written software. Transmission electron microscope (TEM) images of the crystals of dl-arginine and l-arginine are shown in Figs. 1 ▸(*a*) and 1(*c*), respectively, and the corresponding diffraction patterns are shown in Figs. 1 ▸(*b*) and 1(*d*), respectively. The best diffraction showed peaks beyond 0.8 Å. During the experiment, more than 15 data sets were collected from the same grid.

## Data reduction

The data collected from the Jungfrau detector were background corrected and converted to cbf format (Fröjdh *et al.*, 2020 ▸), after which they were indexed, integrated and scaled in *XDS* (Kabsch, 2010 ▸). The data were converted to SHELX HKLF4 format with *XPREP*. The structures were solved *ab initio* using *SHELXT* (Sheldrick, 2015*a* ▸) and subsequent refinement of the structure was done using *SHELXL* (Sheldrick, 2015*b* ▸) and was built in *Shelxle* (Hübschle *et al.*, 2011 ▸). The nine-Cromer–Mann parameter fitting for electron scattering factor was used for *SHELXL* (Prince, 2004 ▸). H atoms were placed automatically when possible (HFIX).

Out of the 15 data sets that were collected from different crystals, only one data set was indexed and integrated with a different unit cell. Structure solution revealed this as the racemic structure of dl-arginine monohydrate. A summary of the data reduction and refinement is presented in Table 1 ▸.

## Structure of the racemic form and anhydrate l-argi­nine

**Figure 2:**
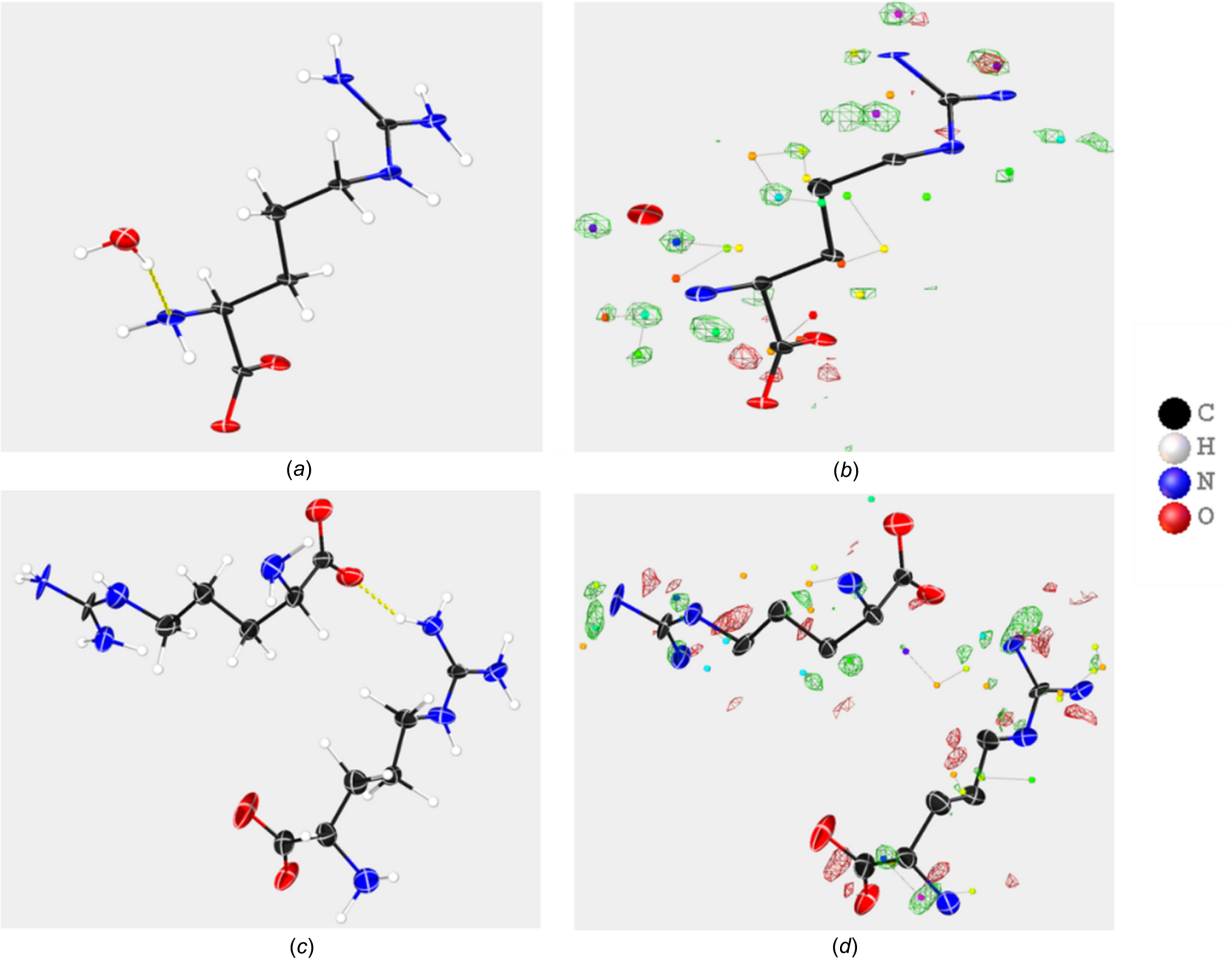
The structural model derived from (*a*) monohydrated dl-arginine, (*b*) the OMIT map of dl-arginine, (*c*) the structural model of l-arginine and (*d*) the OMIT map of l-arginine.

For both the dl-arginine and the l-arginine crystals, the diffraction extended beyond 0.8 Å. The structure of dl-arginine monohydrate shown in Fig. 2 ▸(*a*) is essentially the same as that obtained from X-ray diffraction (Kingsford-Adaboh *et al.*, 2000 ▸). The asymmetric unit consists of one arginine mol­ecule (either d- or l-) and one water mol­ecule. l-Arginine, as shown in Fig. 2 ▸(*c*), consists of two mol­ecules in the asymmetric unit with identical chirality. Since we did not perform dynamical refinement, we did not determine the chirality and assigned both mol­ecules as l-arginine based on the description of the supplier (Merck). Both structures display the characteristic three central –CH_2_– groups of l-arginine. The OMIT map, obtained by removing the H atoms from the model, for both structures are presented in Figs. 2 ▸(*b*) and 2(*d*). In the OMIT maps, one can clearly observe the electrostatic potentials for the H atoms. The results also display that with 3D ED most of the H-atom positions can be correctly assigned with the kinematic refinement. Further improvement in the H-atom position assignment can be done by taking into account the presence of the dynamic scattering effect in the 3D ED data (Clabbers *et al.*, 2019 ▸).

The structures of dl-arginine monohydrate previously solved by SCXRD and solved here by 3D ED show a high degree of similarity. The unit cell obtained by SCXRD (*a* = 11.47, *b* = 9.96 and *c* = 16.0230 Å, and α = β = γ = 90°) is similar to that obtained by 3D ED. Both experiments resulted in the centrosymmetric space group *Pbca*, which means that the sample was racemic. Similarly, the structure of l-arginine solved by 3D ED also shows a high degree of similarity to that solved by PXRD. Both the experiments solved the structure in the space group *P*2_1_ with two mol­ecules in the asymmetric unit. The unit cell of l-arginine solved by PXRD is *a* = 9.75, *b* = 16.02 and *c* = 5.6 Å, and α = γ = 90 and β = 98.05°. The β value obtained by 3D ED is about 3.3% smaller than that obtained by PXRD. The difference can be attributed to the fact that 3D ED determines the unit cell from the single crystal, while PXRD determines an average unit cell from many crystals.

**Figure 3:**
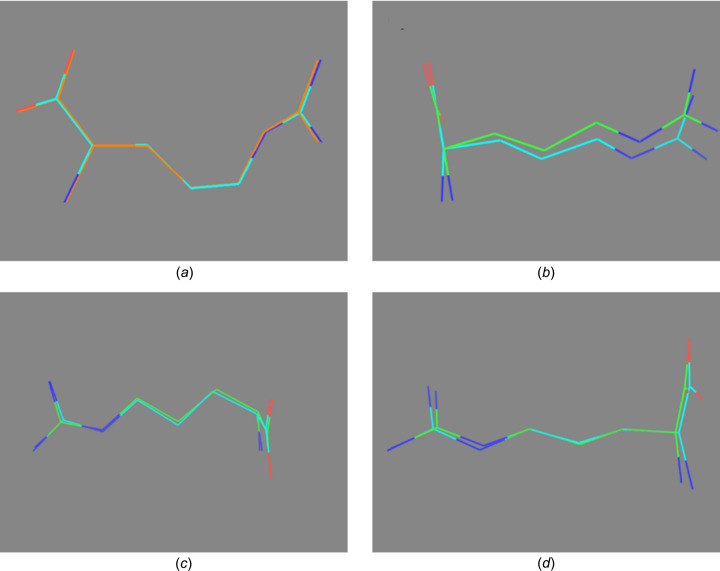
(*a*) Comparison of the model of monohydrated dl-arginine obtained by single-crystal X-ray diffraction and 3D ED. Comparison of the mol­ecules in the asymmetric unit of l-arginine solved by (*b*) PXRD and (*c*) 3D ED, and (*d*) com­parison of the l-arginine mol­ecule solved by PXRD and 3D ED.

Fig. 3 ▸(*a*) shows a com­parison of the monohydrate dl structure solved by 3D ED and that solved from SCXRD. The structures solved by the two different methods align quite well, with an r.m.s. deviation (RMSD) of 0.047 Å. The alignment of the two mol­ecules in the asymmetric unit of l-arginine solved by PXRD and 3D ED are shown in Figs. 3 ▸(*b*) and 3(*c*), respectively. The two mol­ecules in the asymmetric unit of l-arginine align well for the data collected with both PXRD and 3D ED. A com­parison of the l-arginine mol­ecules solved by 3D ED and PXRD is shown in Fig. 3 ▸(*d*). The RMSD between the two structures is 0.083 Å.

The unit cell consists of two mol­ecules of l-arginine. Both mol­ecules display the characteristic three central –CH_2_– groups of l-arginine. The two mol­ecules in l-arginine extend in opposite directions along their respective longitudinal axes. In the racemate, one free water mol­ecule is present in the unit cell. The ability to determine both the hydrated racemate and the anhydrous single enantiomer from the same grid also shows the strength of 3D ED over PXRD. Furthermore, it serves as evidence that the water mol­ecule in the unit cell can be preserved under the high vacuum environment of the transmission electron microscope (TEM), if the samples are cooled under liquid nitro­gen.

During the refinement, 299 parameters were refined for l-arginine. The distance between the N atoms and the H atoms, and the angle between the C atoms and the H atoms were restrained during the refinement. For dl-arginine, 182 parameters were refined and constraints were applied to fix the lengths of the C—H and N—H bonds. In the potential maps of both mol­ecules, we see the H-potential with almost free refinement of the coordinates. These capabilities of 3D ED make it the method of choice in solving structures from nanocrystals where other methods present practical challenges.

## Conclusion

We have determined the single-crystal structure of l-arginine using 3D ED. We also present the structure of two different forms of arginine found in commercially available l-arginine powder, of which the racemic form is a trace amount. The racemic form includes one mol­ecule of water. In the solid state, the structure of l-arginine consists of two mol­ecules in the asymmetric unit. Our work showcases how 3D ED can prove beneficial in determining single-crystal structure from micro/nanocrystals and in the highly sensitive screening of impurities in commercial chemical products.

## Supplementary Material

Crystal structure: contains datablock(s) L-arginine, DL-arginine, global. DOI: 10.1107/S2053229625005091/yp3243sup1.cif

Structure factors: contains datablock(s) L-arginine. DOI: 10.1107/S2053229625005091/yp3243L-argininesup2.hkl

Structure factors: contains datablock(s) DL-arginine. DOI: 10.1107/S2053229625005091/yp3243DL-argininesup3.hkl

CCDC references: 2392312, 2456550

## Figures and Tables

**Table 1:** Summary of the data reduction and structure refinement

3D ED experimental information	L-Arginine	DL-Arginine monohydrate
Collection method	Continuous rotation data collection	Continuous rotation data collection
Number of crystals used for structure determination	1	1
Tilt range	−60 to 70°	−50 to 70°
Tilt increament	0.1°/frame	0.1°/frame
Temperature (°C)	−110	−110
Beam diameter (µm)	2.2	2.2
Camera length (mm)	665	665
Data com­pleteness (%)	88.3	84.8
Data resolution (Å)	0.76	0.73
		
Crystal information		
Empirical formula	C_6_H_14_N_4_O_2_	C_6_H_14_N_4_O_2_·H_2_O
Space group	*P*2_1_	*Pbca*
*a*, *b*, *c* (Å)	5.72 (11), 16.46 (3), 10.05 (2)	11.718 (2), 10.0950 (2), 16.294 (3)
α, β, γ (°)	90.000, 94.83 (3), 90.000	90.000, 90.000, 90.000
*N*_obs_	3556	1478 (2013)
*R*1*	14.62% (20.73%)	18.49% (20.77%)
*R*_int_	0.32	0.146
CCDC ID	2456550	2392312

Note: (*) *R*1 values in parenthesis are for all and values outside parentheses are for *F*_o_ > 4σ(*F*_o_). Computer programs: *SHELXT2018* (Sheldrick, 2015*a* ▸) and *SHELXL2019* (Sheldrick, 2015*b* ▸).

## Data Availability

The data set used for the article is available through 10.5281/zenodo.15550484.
